# Timing the Brain: Mental Chronometry as a Tool in Neuroscience

**DOI:** 10.1371/journal.pbio.0030051

**Published:** 2005-02-15

**Authors:** Michael I Posner

## Abstract

Mental chronometry, which has origins dating back over a century, seeks to measure the time course of mental operations in the human nervous system

How do we relate human thought processes to measurable events in the brain? Mental chronometry, which has origins that date back more than a century, seeks to measure the time course of mental operations in the human nervous system [1]. From the late 1800s until 1950, the field was built almost entirely around a single method: measuring and comparing people's reaction times during simple cognitive tasks. As far back as 1868, Franciscus Donders [2] subtracted the time taken to make a single response to an unvarying stimulus—what he called an instructed reflex—from the time it took to make the same response to one of two events, obtaining the time required to discriminate between the two stimuli. Further, he subtracted the time to discriminate two stimuli from a situation in which there were also two possible responses in order to obtain the time required for choice.

In the 1950s, studies of reaction time were combined with the then-developing mathematical theory of information [3] to address issues such as the maximum transmission rate of the human nervous system [4] and how coding in the brain of stimuli and their responses could influence these limits [5]. These studies revealed that reaction time alone was not sufficient to elucidate the exact processes by which the brain achieved the human ability to process information. However, when combined with other methods, the latency of responding can help connect brain studies to the behavior of humans in real situations.

Recording the average event-related electrical potentials from scalp electrodes became a research tool in the 1960s, with the advent of analog and then digital computers to accomplish the recording and averaging. It became clear that components of the event-related potential could be systematically related to sensory and motor stages of information processing. For instance, a visual stimulus was found to evoke a short-latency scalp response from the primary visual cortex at about 60 milliseconds, followed by positive and negative voltage changes in neighboring visual areas. Similarly, scalp recorded potentials from the frontal cortex could be recorded in relation to motor activity. It was now possible to observe some of the sensory and motor stages that were inferred from Donders's subtractive method (see [6] for a review).

Saul Sternberg [7] developed a much-improved method for dividing reaction time into successive or serial stages, called the additive factors method. Subjects were asked to determine whether or not a probe digit had been present in a just previously presented series of digits. Sternberg argued that the time to complete the task could be divided into a sensory stage, dependent on stimulus parameters such as the intensity or clarity of the probe; a comparison stage, dependent only on the number of items in memory; and a response stage that reflected the difficulty of the specified response. Factors that influenced one stage (e.g., stimulus intensity) would be additive with those that influenced another stage (e.g., motor output). With this simple framework, it was now possible to determine at which stage(s) a new factor (e.g., nicotine, sleep deprivation, or Parkinson's disease) had its influence.

In the 1950s, the advent of microelectrode recordings of single neurons from anesthetized monkeys allowed for an even finer resolution of neurophysiological processes and seemed to provide support for the view that the brain does indeed process information in serial stages. Hubel and Wiesel [8] argued that successive levels of the visual system could be seen as accomplishing successive analyses of input. The microelectrode strategy was quickly adopted to alert animals, making it apparent that higher level brain areas involved in operations upon input might feedback their influences on earlier processing stages [9,10]. These control systems, often called attention, posed something of a problem for completely serial views of information processing. However, they also provided evidence of localized brain areas within the parietal lobe of the monkey that could be systematically related to processing operations involved in attention—which were then being investigated by mental chronometry in patients with parietal and other lesions [11].

In the late 1980s, neuroimaging experiments made possible the examination of activity in localized brain areas, first through the use of injected radionuclides detected by positron emission tomography (PET) [12] and later through the use of an externally imposed magnetic field in functional magnetic resonance imaging (fMRI) [13]. Over the last ten years, fMRI has improved in spatial and temporal resolution and can now provide evidence of quite specific brain areas, in the millimeter range, that are involved in cognitive tasks. Most studies have shown a small number of widely distributed brain areas that must be orchestrated to carry out a cognitive task. Although, as in all sciences, there are disagreements, the convergence of results in areas of attention and language seem to me particularly impressive.

When the fMRI method for localization is brought together with methods that can accurately measure timing of the same activity (i.e., electrical or magnetic event-related potentials) they can provide considerable insight into the nature of thought. Consider the simple task of deciding whether a presented digit is above or below five [14]. Dehaene argued that the task could be divided into four stages. The first involves obtaining the identity of the probe input (encoding), the second making a comparison against the stored representation of the digit five, the third selecting a response, and lastly, checking the output for error. According to additive factor theory, a variable that effects overall reaction time by varying the time to complete one stage will be additive with the effects of variables that affect other stages. The input or encoding stage was varied by using either Arabic or spelled digits (e.g., “3” or “three”). The comparison stage was varied by comparing digits close to five (e.g., six) with those far from five (e.g., nine). The response stage was varied by specifying a response from either the dominant or non-dominant hand and error monitoring was examined by comparing error with correct trials. Each of these variables influenced only the appropriate stage and was additive in its effect with each of the other variables (see Figure 1).

**Figure 1:**
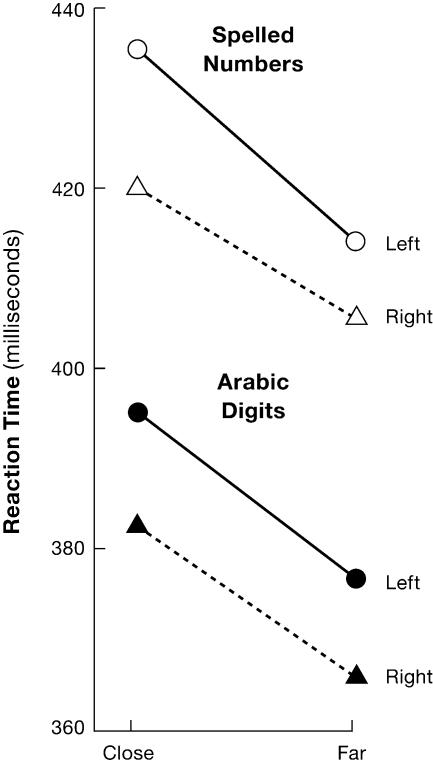
Reaction Time for Various Conditions People were asked to judge whether a presented digit was greater or less than five. The time to respond (reaction time) varied systematically as a function of notation (Arabic digits vs. spelledout numbers), distance (closer or farther in sequence from five), and responding hand. The three effects are additive, indicating the likelihood of serial stages of cognitive processing. (Adapted from [14])

Moreover, scalp-recorded, event-related potentials showed a separate scalp distribution and latency for each variable [14]. Subsequent fMRI data has confirmed and increased the precision of the anatomy proposed for each of these stages.

Of course, not all human activities involve a set of exhaustive and independent serial stages that can be shown to add up to the overall reaction time. While tasks like the number comparison discussed above can be usefully divided into stages, some components may deal with simultaneous operations and may be limited only by a total capacity of central mechanisms. We know that many situations involve parallel processing and feedback loops at many levels. Sternberg has attempted to apply a modified version of additive factor theory to brain systems using neuroimaging that allows for some of these possibilities [15,16].[Fig pbio-0030051-g002]

**Figure 2 pbio-0030051-g002:**
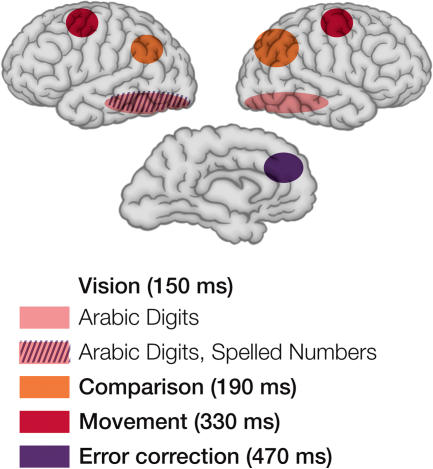
Regions of the Brain Involved in a Number Comparison Task Derived from EEG and fMRI Studies The regions represented correspond to those showing effects of notation used for the numbers (pink and hatched), distance from the test number (orange), choice of hand (red), and errors (purple). (Illustration: Giovanni Maki; adapted from [18])

Laboratory studies often use the simultaneous execution of two different tasks (dual tasks) to simulate the more realistic situations where humans time-share activities. In this issue of *PLoS Biology*, Sigman and Dehaene [17] provide a model that further extends the additive factor logic to the dual task situation. They propose that for tasks that can be broken down into three consecutive stages—perception, decision based on noisy integration of evidence, and response—the perceptual and motor stages can operate both simultaneously with and independently of stages of another task and are thus easily amenable to additive factor analysis. The decision stage, however, appears to represent a kind of “cognitive bottleneck” for which the reaction times of the two tasks become interdependent. The model adds considerably to the range of situations to which an additive factor approach can be applied, allowing investigators to seek more information about how new variables influence hidden processing stages.

Many cognitive and emotional tasks studied with neuroimaging have implicated a small number of brain areas that are consistently active. Mental chronometry plays a role in suggesting the cognitive operations that each of these areas performs and how they are organized in real time. The toolkit of new techniques provides the basis for further tests to evaluate whether a chronometric model reveals a crucial set of connected computations (circuit) for carrying out the task. For example, using a magnetic pulse delivered outside the skull, it is now possible to induce a reversible lesion at the time of a particular computation to determine whether the specific computation assigned to a given area is truly needed to carry out the task. Studies using diffusion tensor imaging can examine whether there are large-scale connections between neural areas posited by a particular model. In describing the links between brain and behavior, mental chronometry is still a cornerstone that binds psychology to the techniques of neuroscience.

## Abbreviations

fMRI: functional magnetic resonance imaging
PET: positron emission tomography
